# Overexpression of epithelial growth factor receptor (EGFR) predicts better response to neo-adjuvant chemotherapy in patients with triple-negative breast cancer

**DOI:** 10.1186/1479-5876-10-S1-S4

**Published:** 2012-09-19

**Authors:** Yiqing Tang, Li Zhu, Yafen Li, Jun Ji, Jianfang Li, Fei Yuan, Dengbin Wang, Weiguo Chen, Ou Huang, Xiaosong Chen, Jiayi Wu, Kunwei Shen, Wings TY Loo, Louis WC Chow

**Affiliations:** 1 Comprehensive Breast Health Center, Ruijin Hospital, School of Medicine, Shanghai Jiaotong University, Shanghai, PRC; 2 Research Institute of General Surgery, Ruijin Hospital, School of Medicine, Shanghai Jiaotong University, Shanghai, PRC; 3 Department of Pathology, Ruijin Hospital, School of Medicine, Shanghai Jiaotong University, Shanghai, PRC; 4 Department of Radiology, Ruijin Hospital, School of Medicine, Shanghai Jiaotong University, Shanghai, PRC; 5 Organisation for Oncology and Translational Research, Hong Kong SAR

## Abstract

**Background:**

Triple negative breast cancer (TNBC) occurs in approximately 10% to 25% of all patients with breast cancer and is associated with poor prognosis. Neo-adjuvant chemotherapy has been reported to produce a higher pathologic complete response (pCR) rate in TNBC. If pCR is achieved, patients with TNBC had a similar survival with non-TNBC patients. The aim of our study was to investigate the protein expression of epithelial growth factor receptor (EGFR) and response to neo-adjuvant chemotherapy and clinical outcome in patients with TNBC compared with non-TNBC.

**Methods:**

A total of 198 locally advanced breast cancer patients who received neo-adjuvant chemotherapy were studied. Immunohistochemistry (IHC) was carried out to detect the protein expression of EGFR in tumor samples. Clinical and pathological parameters, pCR rate and survival data were compared between 40 TNBCs and 158 non-TNBCs.

**Results:**

In 198 cases who received neo-adjuvant chemotherapy, significant differences exist in surgical therapy (P=0.005) and pCR rate (P=0.012) between patients with TNBCs and non-TNBCs. Overexpression of EGFR was significantly associated with pCR rate in patients with TNBCs (P < 0.001). Survival analysis revealed that patients with TNBCs had worse DFS and OS than those with non-TNBCs (P = 0.001, P < 0.001 respectively). Furthermore, for patients with non-TNBCs, those who acheived pCR had better DFS and OS than those who acheived RD (both P < 0.001).

**Conclusions:**

Our results suggested that patients with TNBCs had increased pCR rates compared with non-TNBC. Overexpression of EGFR predicted better response to neo-adjuvant chemotherapy in patients with TNBCs.

## Background

Breast cancer is one of the most frequently diagnosed malignant tumors and the main cause of cancer related death in women worldwide. About 1.38 million new breast cancer cases and 0.46 million breast cancer deaths are estimated to have occurred in 2008 [1]. As the most common malignant tumor of female patients, breast cancer is now recognized as a heterogeneous disease exhibiting substantial differences with regard to biological behavior and requiring distinct therapeutic interventions [2]. Gene expression analysis has revealed five subgroups of breast cancer (luminal A, luminal B, human epidermal growth factor receptor 2 overexpressing, basal like and normal like) by using DNA microarrays. Different subgroups respond differently to therapy and have different outcomes. Survival times of patients with basal-like and human epidermal receptor 2 (HER2)-overexpressing subgroups are the shortest [3-5]. Triple-negative breast cancer (TNBC) is characterized by lacking of expression of both estrogen receptor (ER) and progesterone receptor (PR) as well as HER2 [6,7]. It has some overlap with basal like breast cancer, but the overlap is not complete, because basal like subtype overexpresses myoepithelial cytokeratins (CKs) such as CK 5/6, CK 17 and EGFR [8]. Data were shown that 15%-54% of basal like breast cancers overexpress at least one of ER, PR or HER2 [8-10]. The basal like subtype is identified by DNA microarray, but this method is not available in clinical work readily. The identification of TNBC in clinical work is usually achieved by immunohistochemistry (IHC), which may lead to inclusion of the partial basal-like subtype, inevitably owing to lack of CK 5/6 and EGFR status. That means some TNBCs express EGFR in clinical practice.

EGFR is a member of the ErbB family of receptor tyrosine kinases. This family includes EGFR/ ErbB1/ HER1, ErbB2/ HER2/ Neu, ErbB3/ HER3 and ErbB4/ HER4. These receptors play distinct roles in breast carcinomas [11-13]. EGFR is a kind of transmembrane glycoprotein. EGFR-mediated signal transduction pathways are very extensive and important, and they involved in growth, differentiation, proliferation and anabolism regulation of tumor cells [14]. The roles that EGFR and its ligands play in breast carcinoma are a subject of intensive study and controversy. Some retrospective IHC studies indicated that EGFR overexpression in primary tumors could predict a poor prognosis [15-17], while other studies did not establish such a relationship [18,19].

To date, no guidelines for the treatment of TNBC are published. TNBC is not amenable to hormone therapy or the anti-HER-2 monoclonal antibody for its expression profile, and chemotherapy remains the only possible therapeutic option in the adjuvant or metastatic setting in the TNBC. Neo-adjuvant chemotherapy has been reported to produce a higher pathologic complete response (pCR) rate in TNBC patients than non-TNBC patients [20-23]. If pCR is achieved, patients with TNBC had a similar survival with non-TNBC patients. TNBC patients with residual disease (RD) after neo-adjuvant chemotherapy have worse survival compared with non-TNBC patients [24]. Data from M. D. Anderson Cancer Center showed that about 22% patients with TNBCs could achieved pCR to neo-adjuvant chemotherapy [25]. Thus markers are urged to predict the pCR to neo-adjuvant chemotherapy especially in TNBCs.

The purpose of this study was to detect the EGFR expression in TNBCs as well as the relationship between pathological response rate and clinicopathological parameters.

## Methods

### Patients and treatments

A total of 198 consecutive patients with locally advanced breast cancer, who received neo-adjuvant chemotherapy from January 2004 through December 2008 at Department of Surgery of Ruijin Hospital, were studied retrospectively.

All patients received core needle biopsy before neo-adjuvant chemotherapy was implemented. Eighty patients received CEF [cyclophosphamide (CTX) 600mg/m^2^, epidoxorubicin (EPI) 90mg/m^2^ and fluorouracil (5-FU) 500mg/m^2^] and thirty patients received CMF [CTX 600mg/m^2^, methotrexate (MTX) 40mg/m^2^ and 5-FU 500mg/m^2^]. The other 88 patients received 3-weekly docetaxol 75mg/m^2^ and paclitaxol 175mg/m^2^. Granulocyte colony-stimulating factor (G-CSF) would be given if patients had a less than 2000/mm^2^ leukocyte. Response to neo-adjuvant chemotherapy was evaluated after 2 cycles neo-adjuvant chemotherapy. Neo-adjuvant chemotherapy would be continued for another 1 to 2 cycles if response was evaluated as effectiveness, otherwise operation or radiotherapy would be given. All patients who finished neo-adjuvant chemotherapy received surgery and standard adjuvant chemotherapy, endocrine therapy or radiotherapy.

### Immunohistochemistry (IHC)

Consecutive 4-μm tissue sections were cut from the paraffin blocks of core needle biopsy samples and placed on charged poly-L-lysine-coated slides. Immunohistochemistry was performed using a standard technique of buffer wash and incubation with primary and secondary antibodies using a streptavidin-biotin complex (Dako Corp., CA, USA) and immunoperoxidase with the labeling antigen, diamino-benzidine. Antigen retrieval was performed through proteinase K for 10 minutes. Then the sections were treated with peroxidase-blocking reagent for 20 min, rinsed and treated with 1:100 monoclonal anti-EGFR antibody H11 (Dako Corp., CA, USA) at 4^o^C overnight. Sections were rinsed again and treated for 30 min with visualization reagent solution containing both secondary goat anti-rabbit antibody and horseradish peroxidase linked to a common dextran polymer backbone. After rinsing away the excess visualization reagent, the sections were incubated in diaminobenzidine for 10 min to identify the location of immunoprecipitates. Sections were then counterstained with hematoxylin and eosin, and then mounted in Permount. Immunostaining was interpreted using a bright-field Olympus microscope according to the scoring system of the manufacturer’s instruction. The results were interpreted manually as follows: 0, no membrane staining; 1+, faint, partial membrane staining; 2+, weak, complete membrane staining in <10% of invasive cancer cells; 3+, intense complete membrane staining in >10% of invasive cancer cells. Scores of 2+ and 3+ were considered as overexpression and scores of 0 and 1+ were considered as low expression [26]. Controls without primary antibody and positive control tissue were included in all experiments to ensure the staining quality.

### Pathological and clinical response evaluation

ER, PR and HER2 status were assessed by IHC. ER and PR positivity were defined as no less than 10% positive tumor cells with nuclear staining and HER2 positivity was defined as 3+ by IHC. Tumors negative for ER, PR and HER2 were classified as TNBCs and tumors with any receptor positivity were classified as non-TNBCs.

Response to neo-adjuvant chemotherapy was evaluated after 2 cycles neo-adjuvant chemotherapy. Clinical effects were evaluated through response evaluation criteria in solid tumor (RECIST 1.0 [14]) instituted by European Organization for Research and Treatment of Cancer (EORTC), National Cancer Institute (NCI) and National Cancer Institute of Canada (NCIC) in 1998. Pathological CR was determined by microscopic examination of the excised tumor and lymph nodes after the completion of neo-adjuvant chemotherapy and it was defined as no residual invasive cancer in tumor or lymph nodes. Patients with carcinoma in situ without any invasive component were also considered as pCR. All the pathological sections which were considered to be pCR were evaluated by another pathologist. pCR was achieved if they both confirmed.

### Statistical analyses

Statistical analysis was performed using the SPSS 17.0 (SPSS, Inc., Chicago, IL). Difference between TNBCs and non-TNBCs with clinicopathological variables were evaluated using the chi-square test. Correlations between pathologic response and clinicopathological characteristics in patients with TNBCs were analysed by logistic regression. Overall survival (OS) was calculated from the date of patients being confirmed as breast carcinoma to the date of last follow-up or death. Distant free survival (DFS) was defined as the period from the date of confirmed diagnosis to the date of last follow-up or metastatic diseases. Survival analysis was using the log-rank test, and survival plots were created using Kaplan-Meier methods. All P values reported were two-sided with P < 0.05 considered to be statistically significant.

## Results

### Patient characteristics

In this retrospective study, a total of 198 consecutive patients aged from 31 to 79 years old were enrolled. Forty (20.20%) cases were designated as TNBCs and 158 (79.80%) patients were non-TNBCs. There was no significant difference (P = 0.118) between mean age of TNBCs and non-TNBCs . Number of TNBCs who received standard radical mastectomy was significantly more than non-TNBCs (P= 0.005). The differences in menstrual status, histology type, tumor stage and expression status of EGFR were not statistically significant (Table 1-2). Pathological CR rate of TNBCs was significantly higher than that of non-TNBCs (P = 0.012, Table 2).

**Table 1:** Correlations between Triple Negative Status and Prognostic Factors

**Factors**	**TNBC**	**Non-TNBC**	P^*^
		
	No. of Patients	No. of Patients	
**Age, years**			
≤35	0	2	1.000
>35	40	156	
**Menstrual Status**			
Postmenopause	16	86	0.103
Premenopause	24	72	
**Surgical therapy**			
Modified Radical Mastectomy and Simple Mastectomy	34	154	0.005
Standard Radical Mastectomy	6	4	

Tables notes:

TNBC means triple-negative breast cancer

*Determined by logistic analysis

**Table 2:** Correlations between Triple Negative Status and Pathological Factors

**Factors**	**TNBC**	**Non-TNBC**	P^*^
		
	No. of Patients	No. of Patients	
**Histology**			
Ductal	38	144	0.534
Nonductal	2	14	
**Prechemotherapy T stage**			
T1	0	10	0.218
T2- T4	40	148	
**Prechemotherapy N stage**			
N0	14	46	0.469
N1- N3	26	112	
**Histological grade**			
I - II	18	78	0.622
III	22	80	
**EGFR**			
Positive	10	28	0.296
Negative	30	130	
**Pathologic Response**			
pCR	10	14	0.012
RD	30	144	

Tables notes:

TNBC: triple-negative breast cancer

*Determined by logistic analysis

### Patients’ outcome

After a median follow-up of 25 months (3 to 58 months), twenty distant metastatic diseases and 12 deaths occurred in all patients. Six patients with TNBCs had distant metastasis and 2 patients with TNBCs died of breast cancer.

### Correlations between pathologic response and clinicopathological characteristics in patients with TNBCs

Overexpression of EGFR was significantly associated with pCR to neo- adjuvant chemotherapy in patients with TNBCs (OR = 59.18, 95% CI 3.77- 927.97), P < 0.001). Pathological CR rate was not significantly related with age, menstrual status, histology type and preoperative tumor stage (P> 0.05, Table 3). But EGFR expression was not an independent predictor of chemotherapeutic response by multivariate analysis (P = 0.714, Table 3).

**Table 3:** Correlations between Pathologic Response and Clinicopathological Characteristics in Patients with TNBCs

Factors	pCR	RD	OR^‡^	P^*^	P^#^
			
	No. of Patients	No. of Patients	(95% CI^†^)		
**Age, years**					
≤35	0	0	/	/	/
>35	10	30	/		
**Menstrual Status**					
Postmenopause	2	14	0.38	0.263	1.000
Premenopause	8	16	(0.09 - 1.54)		
**Histology**					
Ductal	10	28	1.62	1.000	1.000
Nonductal	0	2	(0.12 - 21.37)		
**Prechemotherapy N stage**					
N0	6	20	0.81	0.718	1.000
N1- N3	4	10	(0.27 - 2.39)		
**Histological grade**					
I - II	6	12	1.83	0.300	1.000
III	4	18	(0.61- 5.51)		
**EGFR**					
Positive	10	0	59.18	< 0.001	0.714
Negative	0	30	(3.77- 927.97)		

Table notes:

‡OR: odds ratio

†CI: confidence interval

*Determined by logistic analysis

#Determined by multivariate analysis

### Survival analysis

**Figure 1:**
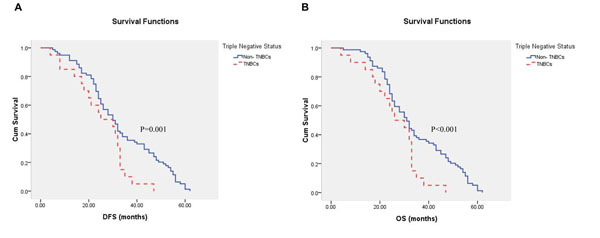
Probability of distant-free survival (DFS) (A) and overall survival (OS) (B) in triple negative breast cancers (TNBCs) and non-TNBCs.

Patients with TNBCs had a worse prognosis than those with non-TNBCs: DFS (mean 25.15 months vs. 33.10 months, P=0.001), OS (mean 26.20 months vs. 34.27 months, P < 0.001) [Figure 1 (A-B)].

**Figure 2:**
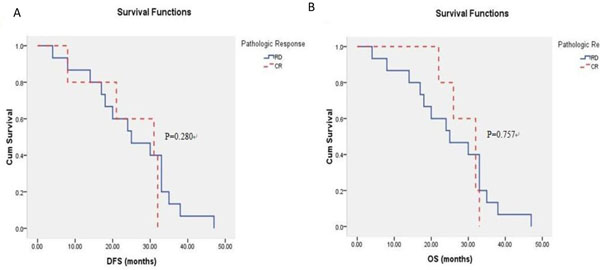
Probability of distant-free survival (DFS) (A) and overall survival (OS) (B) in pathologic complete response (pCR) and residual disease (RD) patients with triple negative breast cancers (TNBCs).

For patients with TNBCs, prognosis between those who achieved pCR to neo-adjuvant chemotherapy and those who just achieved RD had no significant difference: DFS (mean 24.80 months vs. 25.27 months, P=0.280), OS (mean 29.00 months vs. 25.27 months, P=0.757) [Figure 2 (A-B)].

**Figure 3:**
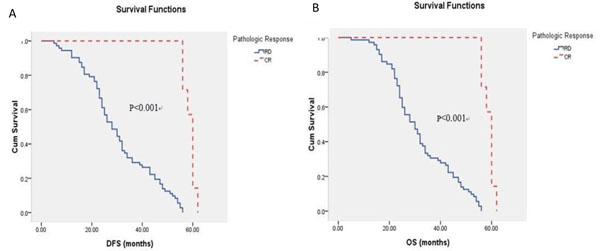
Probability of distant-free survival (DFS) (A) and overall survival (OS) (B) in pathologic complete response (pCR) and residual disease (RD) patients with non-triple negative breast cancers (non-TNBCs).

While for patients with non-TNBCs, those who achieved pCR had significantly better prognosis than those who only achieved RD: DFS (mean 58.86 months vs. 30.60 months, P < 0.001), OS (mean 58.86 months vs. 31.88 months, P < 0.001) [Figure 3 (A-B)].

## Discussion

Breast cancer has been recognized as a heterogeneous disease increasingly. Five subtypes of breast cancer have been revealed by gene expression analysis Nevertheless the methodology is not available readily in clinical practice, so practical and feasible method is to look for immunohistochemical markers for basal like subtype. To date, it is most appropriate to define ER, PR, HER2, CK 5/6 and EGFR as the immunohistochemical markers for basal like subtype [8], which implied that triple negative was the main feature of basal like breast cancer for a lack of expression of ER, PR and HER2 by IHC. Fifty percent to eighty-five percent of TNBCs were reported to be basal like breast cancers [27-29]. So TNBC was not the absolute substitute of basal like subtype. TNBCs may include partial basal like subtype inevitably in clinical practice for lack of CK 5/6, CK 17 and EGFR status.

Different subtypes respond differently to therapy and have different outcomes. Survival times of patients with basal like breast cancer and HER2-overexpressing subtypes are the shortest [3-5]. In this study, data indicated that the DFS and OS of patients with TNBCs were significantly shorter than those with non-TNBCs (P = 0.001, P < 0.001).

TNBC is not amenable to hormone therapy or the anti-HER2 monoclonal antibody for its expression profile, and chemotherapy remains the only possible therapeutic option in the adjuvant or metastatic setting in the TNBC. As a new strategic therapy in recent years, neo-adjuvant therapy represented the conversion of therapeutic philosophy to breast cancer, which was to convert traditional local treatments to paying more attention to systemic treatments. Neo-adjuvant chemotherapy made it possible to be excised for local advanced breast cancer, and also enabled breast conserving surgery in spite of larger tumor size. Subclinical metastatic diseases could be controlled through neo-adjuvant chemotherapy which could improve the survival of patients. But because of a kind of heterologous tumor and its resistance to chemotherapy regimens, controversies still existed, especially for triple negative breast cancer. Neo-adjuvant chemotherapy has been reported to produce a higher pathologic complete response (pCR) rate in TNBC patients than non-TNBC patients [20-23]. Our data indicated that pCR rate was significantly higher in TNBCs compared with non-TNBCs (P=0.012). Patients with non-TNBCs who achieved pCR were proved to have excellent survival compared with those who just achieved RD (P < 0.001). However, difference in survival between patients with TNBCs who achieved pCR and those who achieved RD had no significance (P=0.280, P=0.757). TNBCs have been proved to have poorer prognostic features in general compared with non-TNBCs. In the other hand, chemotherapy is the only systemic treatment option for patients with TNBCs, nevertheless patients with non-TNBCs can benefit from endocrine therapy even targeted therapies such as trastuzumab besides chemotherapy. So patients with non-TNBCs in our study showed better survival. Another reason for no survival benefit for patients who achieved pCR in TNBC group might be due to the small proportion of TNBC in the study cohort.

Research on factors about response to neo-adjuvant chemotherapy appeared to be important. Factors which were studied more included ER, HER2/neu, P53, Ki67 etc. Conclusions were reached differently on account of differences in sample volume and research methods, which leaded to no exact and effective factors to guide clinical work. Proliferation of tumor, histologic grade and ER status appeared to be related to response to neo-adjuvant chemotherapy on the whole. EGFR is a kind of transmembrane glycoprotein. EGFR-mediated signal transduction pathways are very extensive and important, and they involved in growth, differentiation, proliferation and anabolism regulation of tumor cells. Many studies have proved that expression of EGFR in breast carcinoma is significantly higher than in normal epithelial tissue. Our result indicated that EGFR was an important maker of pathological response rate to neo-adjuvant chemotherapy. The overexpression of EGFR indicated more probability to pCR (OR = 59.18, P<0.001). *In vitro* studies on effects of EGFR inhibition in triple negative breast cancer cell lines revealed that gefitinib inhibited EGFR phosphorylation, which led to reduced signaling by the mitogen activated protein kinase (MAPK) and Akt pathway and causing cell cycle arrest at G1 phase [30]. In addition, gefitinib enhanced chemotherapeutic response to both carboplatin and docetaxel in these cells. In a Phase II trial of erlotinib in patients with advanced breast cancer, 2 of 69 patients had partial responses, one of which had triple-negative histology [31].

## Conclusions

In conclusion, we have shown that patients with TNBCs have increased pCR rates compared with non-TNBC. Despite the limited size of the cohort and immature survival data, our finding that EGFR overexpression has predictive value for better response to neo-adjuvant chemotherapy in patients with TNBCs.

## Competing interests

The authors have declared that no conflict of interest exists.

## Authors' contributions

LZ conceived of the study, participated in its design and helped to draft the manuscript. KS participated in its design and co-ordination. YL, WC performed treatments for patients in the study. DW evaluated the clinical stage and response to neoadjuvant chemotherapy of patients in the study through radiation imaging methods. JJ and FY carried out the histopathological analysis and immunohistochemistry. OH, XC and JW participated in data collecting and statistical analysis. YT participated in statistical analysis and manuscript writing. WTYL and LWCC participated in manuscript writing. All authors read and approved the final manuscript.
